# (*E*)-3-[2,5-Dioxo-3-(propan-2-yl­idene)pyrrolidin-1-yl]acrylic acid

**DOI:** 10.1107/S1600536810005040

**Published:** 2010-02-13

**Authors:** Fang Miao, Bao-Fu Qin, Li-Zhen Yang, Xin-Juan Yang, Le Zhou

**Affiliations:** a College of Life Science, Northwest A&F University, Yangling 712100, People’s Republic of China; b College of Science, Northwest A&F University, Yangling 712100, People’s Republic of China

## Abstract

The title compound, C_10_H_11_NO_4_, was extracted from a culture broth of *Penicillium verruculosum* YL-52. The mol­ecular structure is essentially planar, with an r.m.s. deviation of 0.01342 (2) Å for the non-H atoms. In the crystal structure, adjacent mol­ecules are connected into a centrosymmetric dimer through a pair of O—H⋯O hydrogen bonds. The dimers are further extended into a chain by weak C—H⋯O hydrogen bonds.

## Related literature

For a related structure, see: Cheng *et al.* (2009 ▶). For details of *Penicillium verruculosum* YL-52, see: Yang *et al.* (2009 ▶).
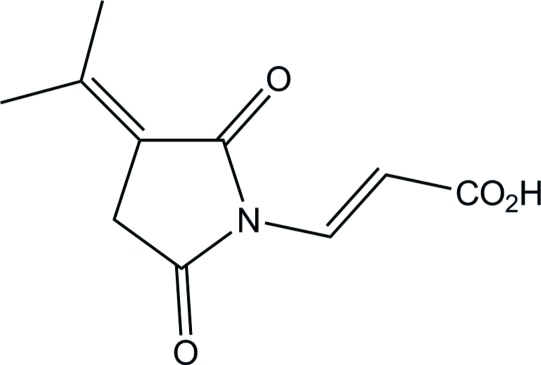


## Experimental

### 

#### Crystal data

C_10_H_11_NO_4_
*M* *_r_* = 209.20
Triclinic, 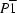

*a* = 6.5384 (14) Å
*b* = 7.5309 (17) Å
*c* = 10.405 (2) Å
α = 93.009 (3)°
β = 101.247 (2)°
γ = 90.410 (3)°
*V* = 501.74 (19) Å^3^
*Z* = 2
Mo *K*α radiation
μ = 0.11 mm^−1^
*T* = 296 K
0.33 × 0.12 × 0.08 mm

#### Data collection

Bruker APEXII CCD diffractometer
Absorption correction: multi-scan (*SADABS*; Sheldrick, 1996 ▶) *T*_min_ = 0.965, *T*_max_ = 0.991
3845 measured reflections
1849 independent reflections
1255 reflections with *I* > 2σ(*I*)
*R*_int_ = 0.024

#### Refinement

*R*[*F* ^2^ > 2σ(*F* ^2^)] = 0.047
*wR*(*F* ^2^) = 0.125
*S* = 1.05
1849 reflections
139 parameters
H-atom parameters constrained
Δρ_max_ = 0.17 e Å^−3^
Δρ_min_ = −0.19 e Å^−3^

### 

Data collection: *APEX2* (Bruker, 2004 ▶); cell refinement: *SAINT* (Bruker, 2004 ▶); data reduction: *SAINT*; program(s) used to solve structure: *SHELXS97* (Sheldrick, 2008 ▶); program(s) used to refine structure: *SHELXL97* (Sheldrick, 2008 ▶); molecular graphics: *SHELXTL* (Sheldrick, 2008 ▶) and *DIAMOND* (Brandenburg, 2006 ▶); software used to prepare material for publication: *SHELXTL* and *PLATON* (Spek, 2009 ▶).

## Supplementary Material

Crystal structure: contains datablocks global, I. DOI: 10.1107/S1600536810005040/is2521sup1.cif

Structure factors: contains datablocks I. DOI: 10.1107/S1600536810005040/is2521Isup2.hkl

Additional supplementary materials:  crystallographic information; 3D view; checkCIF report

## Figures and Tables

**Table 1:** Hydrogen-bond geometry (Å, °)

*D*—H⋯*A*	*D*—H	H⋯*A*	*D*⋯*A*	*D*—H⋯*A*
O1—H1⋯O2^i^	0.82	1.83	2.647 (2)	174
C6—H6*A*⋯O4^ii^	0.97	2.60	3.399 (3)	140

Symmetry codes: (i) 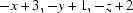; (ii) 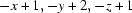.

**Fig. 1.**
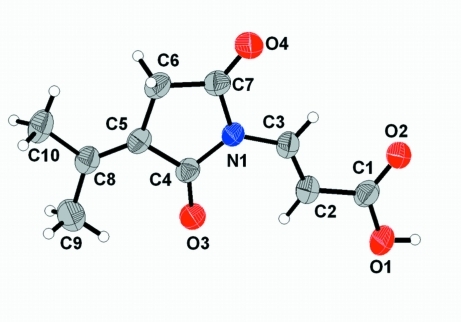
View of the title molecular structure with atom numbering scheme and 30% probability displacement ellipsoids for non-hydrogen atoms.

**Fig. 2.**
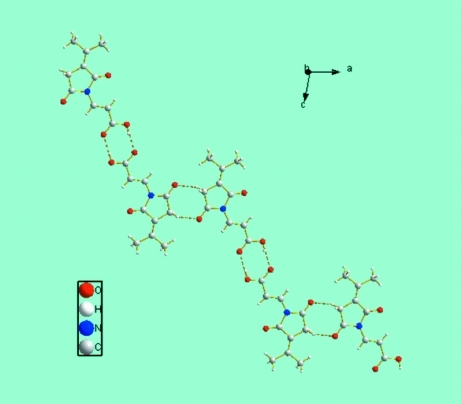
View of the two-dimensional sheet (O—H···O and C—H···O hydrogen bonds are indicated as broken lines).

## supplementary crystallographic information

### Comment

Stellera chamaejasme L belongs to a toxic plant and its root has been used as Chinese traditional herb medicine in China. Our previous study resulted in isolating a fungal strain from the rhizosphere of *Stellera Chamaejasme* L identified as *Penicillium verruculosum* YL-52 (Yang *et al.*, 2009). In this controbution, we reported the crystal structure of the title compound which was obtained from the culture broth of *Penicillium verruculosum* YL-52.

The bond lengths and angles of the title compound are within normal ranges (Cheng *et al.*, 2009). In the crystal structure, the molecule, excluding methyl H atoms, is essentially planar, with an r.m.s. deviation of 0.01342 (2) Å. Moreover, adjacent two molecules are connected into a dimer through two head to head O1—H1···O2 hydrogen bonds. The dimers are further extended into a one-dimensional chain by weak C—H···O hydrogen bonds along the *b* axis, in which C6—H6A is donor and O4 is acceptor (Table 1 and Fig. 2).

### Experimental

The roots of *Stellera Chamaejasme* L was collected in Qinling mountain of Taibai town in Shaanxi province, P. R. China, in August, 2007, and the fungal strain was isolated from the rhizosphere of the plant above, and deposited in our laboratory of natural product research, Northwest A&F University, Shaanxi Province, the People's Republic of China (culture collection number YL-52), and identified as *Penicillium verruculosum* YL-52. Repeated column chromatography of ethyl acetate extract of the culture broth of *Penicillium verruculosum* YL-52 provided the title compound.

### Refinement

All H atoms were positioned geometrically and treated as riding, with C—H bond lengths constrained to 0.93 Å (CH), 0.97 Å (CH_2_) and 0.96 Å (CH_3_), and with *U*_iso_(H) = 1.2*U*_eq_(C) or 1.5*U*_eq_(methyl C).

### Crystal data

**Table:**

C_10_H_11_NO_4_	*Z* = 2
*M**_r_* = 209.20	*F*(000) = 220
Triclinic, *P*1	*D*_x_ = 1.385 Mg m^−^^3^
Hall symbol: -P 1	Mo *K*α radiation, λ = 0.71073 Å
*a* = 6.5384 (14) Å	Cell parameters from 773 reflections
*b* = 7.5309 (17) Å	θ = 0.00–0.00°
*c* = 10.405 (2) Å	µ = 0.11 mm^−^^1^
α = 93.009 (3)°	*T* = 296 K
β = 101.247 (2)°	Block, colourless
γ = 90.410 (3)°	0.33 × 0.12 × 0.08 mm
*V* = 501.74 (19) Å^3^	

### Data collection

**Table:**

Bruker APEXII CCD diffractometer	1849 independent reflections
Radiation source: fine-focus sealed tube	1255 reflections with *I* > 2σ(*I*)
graphite	*R*_int_ = 0.024
φ and ω scans	θ_max_ = 25.5°, θ_min_ = 2.7°
Absorption correction: multi-scan (*SADABS*; Sheldrick, 1996)	*h* = −7→7
*T*_min_ = 0.965, *T*_max_ = 0.991	*k* = −9→9
3845 measured reflections	*l* = −12→12

### Refinement

**Table:**

Refinement on *F*^2^	Primary atom site location: structure-invariant direct methods
Least-squares matrix: full	Secondary atom site location: difference Fourier map
*R*[*F*^2^ > 2σ(*F*^2^)] = 0.047	Hydrogen site location: inferred from neighbouring sites
*wR*(*F*^2^) = 0.125	H-atom parameters constrained
*S* = 1.05	*w* = 1/[σ^2^(*F*_o_^2^) + (0.0581*P*)^2^ + 0.0541*P*] where *P* = (*F*_o_^2^ + 2*F*_c_^2^)/3
1849 reflections	(Δ/σ)_max_ < 0.001
139 parameters	Δρ_max_ = 0.17 e Å^−^^3^
0 restraints	Δρ_min_ = −0.19 e Å^−^^3^

### Special details

**Table:**

Geometry. All esds (except the esd in the dihedral angle between two l.s. planes) are estimated using the full covariance matrix. The cell esds are taken into account individually in the estimation of esds in distances, angles and torsion angles; correlations between esds in cell parameters are only used when they are defined by crystal symmetry. An approximate (isotropic) treatment of cell esds is used for estimating esds involving l.s. planes.
Refinement. Refinement of *F*^2^ against ALL reflections. The weighted *R*-factor wR and goodness of fit *S* are based on *F*^2^, conventional *R*-factors *R* are based on *F*, with *F* set to zero for negative *F*^2^. The threshold expression of *F*^2^ > σ(*F*^2^) is used only for calculating *R*-factors(gt) etc. and is not relevant to the choice of reflections for refinement. *R*-factors based on *F*^2^ are statistically about twice as large as those based on *F*, and *R*-factors based on ALL data will be even larger.

### Fractional atomic coordinates and isotropic or equivalent isotropic displacement parameters (Å^2^)

**Table:**

	*x*	*y*	*z*	*U*_iso_*/*U*_eq_	
O2	1.2795 (3)	0.5891 (2)	0.92347 (15)	0.0617 (5)	
O1	1.5542 (3)	0.5091 (3)	0.83867 (17)	0.0664 (5)	
H1	1.5982	0.4818	0.9142	0.100*	
N1	0.9689 (2)	0.7432 (2)	0.55825 (16)	0.0375 (4)	
O4	0.6865 (2)	0.8327 (2)	0.64451 (17)	0.0611 (5)	
O3	1.2017 (2)	0.6881 (2)	0.42194 (15)	0.0555 (5)	
C4	1.0334 (3)	0.7417 (3)	0.4361 (2)	0.0372 (5)	
C3	1.0819 (3)	0.6860 (3)	0.6763 (2)	0.0403 (5)	
H3	1.0143	0.6904	0.7472	0.048*	
C5	0.8620 (3)	0.8155 (3)	0.3422 (2)	0.0382 (5)	
C8	0.8650 (3)	0.8428 (3)	0.2160 (2)	0.0445 (6)	
C2	1.2744 (3)	0.6264 (3)	0.6983 (2)	0.0437 (6)	
H2	1.3491	0.6184	0.6309	0.052*	
C1	1.3693 (3)	0.5730 (3)	0.8297 (2)	0.0441 (6)	
C6	0.6859 (3)	0.8529 (3)	0.4118 (2)	0.0458 (6)	
H6A	0.6463	0.9765	0.4060	0.055*	
H6B	0.5654	0.7782	0.3743	0.055*	
C7	0.7675 (3)	0.8117 (3)	0.5505 (2)	0.0433 (5)	
C10	0.6796 (4)	0.9171 (3)	0.1284 (2)	0.0580 (7)	
H10A	0.5714	0.9396	0.1773	0.087*	
H10B	0.6294	0.8331	0.0563	0.087*	
H10C	0.7192	1.0262	0.0953	0.087*	
C9	1.0460 (4)	0.8041 (4)	0.1519 (3)	0.0675 (8)	
H9A	1.1076	0.9138	0.1338	0.101*	
H9B	0.9992	0.7347	0.0713	0.101*	
H9C	1.1476	0.7388	0.2093	0.101*	

### Atomic displacement parameters (Å^2^)

**Table:**

	*U*^11^	*U*^22^	*U*^33^	*U*^12^	*U*^13^	*U*^23^
O2	0.0608 (11)	0.0854 (13)	0.0385 (10)	0.0281 (9)	0.0066 (8)	0.0098 (8)
O1	0.0544 (11)	0.0958 (14)	0.0481 (10)	0.0294 (10)	0.0024 (8)	0.0214 (10)
N1	0.0320 (9)	0.0440 (10)	0.0355 (10)	0.0051 (8)	0.0029 (8)	0.0055 (8)
O4	0.0491 (10)	0.0836 (12)	0.0550 (11)	0.0187 (9)	0.0177 (9)	0.0121 (9)
O3	0.0375 (9)	0.0838 (12)	0.0452 (9)	0.0178 (8)	0.0060 (7)	0.0105 (8)
C4	0.0296 (11)	0.0413 (12)	0.0396 (12)	0.0026 (9)	0.0032 (9)	0.0046 (9)
C3	0.0427 (13)	0.0417 (12)	0.0348 (12)	0.0027 (10)	0.0024 (10)	0.0065 (9)
C5	0.0328 (11)	0.0386 (12)	0.0413 (13)	0.0022 (9)	0.0014 (9)	0.0054 (9)
C8	0.0448 (13)	0.0447 (13)	0.0414 (13)	0.0022 (10)	0.0009 (10)	0.0051 (10)
C2	0.0446 (14)	0.0478 (13)	0.0373 (12)	0.0043 (11)	0.0027 (10)	0.0072 (10)
C1	0.0426 (13)	0.0442 (13)	0.0425 (13)	0.0081 (10)	0.0005 (11)	0.0045 (10)
C6	0.0360 (12)	0.0521 (13)	0.0480 (14)	0.0092 (10)	0.0033 (10)	0.0093 (11)
C7	0.0364 (12)	0.0478 (13)	0.0465 (14)	0.0048 (10)	0.0094 (11)	0.0055 (10)
C10	0.0571 (16)	0.0625 (16)	0.0484 (15)	0.0082 (12)	−0.0073 (12)	0.0138 (12)
C9	0.0633 (17)	0.093 (2)	0.0487 (15)	0.0124 (15)	0.0150 (13)	0.0130 (14)

### Geometric parameters (Å, °)

**Table:**

O2—C1	1.234 (3)	C8—C9	1.491 (3)
O1—C1	1.293 (3)	C8—C10	1.499 (3)
O1—H1	0.8200	C2—C1	1.465 (3)
N1—C3	1.395 (3)	C2—H2	0.9300
N1—C7	1.407 (3)	C6—C7	1.488 (3)
N1—C4	1.415 (3)	C6—H6A	0.9700
O4—C7	1.203 (3)	C6—H6B	0.9700
O3—C4	1.207 (2)	C10—H10A	0.9600
C4—C5	1.469 (3)	C10—H10B	0.9600
C3—C2	1.321 (3)	C10—H10C	0.9600
C3—H3	0.9300	C9—H9A	0.9600
C5—C8	1.343 (3)	C9—H9B	0.9600
C5—C6	1.496 (3)	C9—H9C	0.9600
			
C1—O1—H1	109.5	C7—C6—C5	105.09 (17)
C3—N1—C7	120.90 (18)	C7—C6—H6A	110.7
C3—N1—C4	127.07 (17)	C5—C6—H6A	110.7
C7—N1—C4	112.03 (17)	C7—C6—H6B	110.7
O3—C4—N1	122.39 (18)	C5—C6—H6B	110.7
O3—C4—C5	130.8 (2)	H6A—C6—H6B	108.8
N1—C4—C5	106.80 (17)	O4—C7—N1	122.9 (2)
C2—C3—N1	127.1 (2)	O4—C7—C6	129.1 (2)
C2—C3—H3	116.5	N1—C7—C6	107.93 (18)
N1—C3—H3	116.5	C8—C10—H10A	109.5
C8—C5—C4	125.4 (2)	C8—C10—H10B	109.5
C8—C5—C6	126.63 (19)	H10A—C10—H10B	109.5
C4—C5—C6	107.97 (18)	C8—C10—H10C	109.5
C5—C8—C9	124.3 (2)	H10A—C10—H10C	109.5
C5—C8—C10	120.9 (2)	H10B—C10—H10C	109.5
C9—C8—C10	114.8 (2)	C8—C9—H9A	109.5
C3—C2—C1	119.5 (2)	C8—C9—H9B	109.5
C3—C2—H2	120.2	H9A—C9—H9B	109.5
C1—C2—H2	120.2	C8—C9—H9C	109.5
O2—C1—O1	123.3 (2)	H9A—C9—H9C	109.5
O2—C1—C2	122.4 (2)	H9B—C9—H9C	109.5
O1—C1—C2	114.3 (2)		
			
C3—N1—C4—O3	−0.1 (3)	C6—C5—C8—C10	−0.4 (3)
C7—N1—C4—O3	179.89 (19)	N1—C3—C2—C1	179.57 (19)
C3—N1—C4—C5	−179.71 (17)	C3—C2—C1—O2	−3.5 (3)
C7—N1—C4—C5	0.3 (2)	C3—C2—C1—O1	177.0 (2)
C7—N1—C3—C2	−176.9 (2)	C8—C5—C6—C7	−176.1 (2)
C4—N1—C3—C2	3.1 (3)	C4—C5—C6—C7	4.3 (2)
O3—C4—C5—C8	−2.1 (4)	C3—N1—C7—O4	3.7 (3)
N1—C4—C5—C8	177.4 (2)	C4—N1—C7—O4	−176.4 (2)
O3—C4—C5—C6	177.6 (2)	C3—N1—C7—C6	−177.54 (18)
N1—C4—C5—C6	−2.9 (2)	C4—N1—C7—C6	2.4 (2)
C4—C5—C8—C9	−0.8 (4)	C5—C6—C7—O4	174.6 (2)
C6—C5—C8—C9	179.6 (2)	C5—C6—C7—N1	−4.1 (2)
C4—C5—C8—C10	179.3 (2)		

### Hydrogen-bond geometry (Å, °)

**Table:**

*D*—H···*A*	*D*—H	H···*A*	*D*···*A*	*D*—H···*A*
O1—H1···O2^i^	0.82	1.83	2.647 (2)	174
C6—H6A···O4^ii^	0.97	2.60	3.399 (3)	140

Symmetry codes: (i) −*x*+3, −*y*+1, −*z*+2; (ii) −*x*+1, −*y*+2, −*z*+1.
